# Association between XRCC1 and XRCC3 Polymorphisms with Lung Cancer Risk: A Meta-Analysis from Case-Control Studies

**DOI:** 10.1371/journal.pone.0068457

**Published:** 2013-08-26

**Authors:** Guohua Huang, Shaoxi Cai, Wei Wang, Qing Zhang, Aihua Liu

**Affiliations:** 1 Department of Respiration, Nanfang Hospital of Southern Medical University, Guangzhou, China; 2 Gastroenterology Department, The Second People's Hospital of Zhuhai, Zhuhai, China; 3 Beijing Zhendong Guangming Pharmaceutical Research Institute Co. Ltd., Beijing, China; 4 Shanxi Zhendong Pharmaceutical Co. Ltd., Changzhi, China; 5 Department of Pharmacy, Nanfang Hospital of Southern Medical University, Guangzhou, China; MOE Key Laboratory of Environment and Health, School of Public Health, Tongji Medical College, Huazhong University of Science and Technology, China

## Abstract

Many studies have reported the association of X-ray repair cross-complementing group 1 (*XRCC1*) Arg399Gln, Arg194Trp, Arg280His, −77T>C, and X-ray repair cross-complementing group 3 (*XRCC3*) T241M polymorphisms with lung cancer risk, but the results remained controversial. Hence, we performed a meta-analysis to investigate the association between lung cancer risk and *XRCC1* Arg399Gln (14,156 cases and 16,667 controls from 41 studies), Arg194Trp (7,426 cases and 9,603 controls from 23 studies), Arg280His (6,211 cases and 6,763 controls from 16 studies), −77T>C (2,487 cases and 2,576 controls from 5 studies), and *XRCC3* T241M (8,560 cases and 11,557 controls from 19 studies) in different inheritance models. We found that −77T>C polymorphism was associated with increased lung cancer risk (dominant model: odds ration [OR] = 1.45, 95% confidence interval [CI] = 1.27–1.66, recessive model: OR = 1.73, 95% CI = 1.14–2.62, additive model: OR = 1.91, 95% CI = 1.24–1.94) when all the eligible studies were pooled into the meta-analysis. In the stratified and sensitive analyses, significantly decreased lung cancer risk was observed in overall analysis (dominant model: OR = 0.83, 95% CI = 0.78–0.89; recessive model: OR = 0.90, 95% CI = 0.81–1.00; additive model: OR = 0.82, 95% CI = 0.74–0.92), Caucasians (dominant model: OR = 0.82, 95% CI = 0.76–0.87; recessive model: OR = 0.89, 95% CI = 0.80–0.99; additive model: OR = 0.81, 95% CI = 0.73–0.91), and hospital-based controls (dominant model: OR = 0.81, 95% CI = 0.76–0.88; recessive model: OR = 0.89, 95% CI = 0.79–1.00; additive model: OR = 0.80, 95% CI = 0.71–0.90) for *XRCC3* T241M. In conclusion, this meta-analysis indicates that *XRCC1* −77T>C shows an increased lung cancer risk and *XRCC3* T241M polymorphism is associated with decreased lung cancer risk, especially in Caucasians.

## Introduction

Lung cancer is a major cause of cancer-related death in the worldwide and the overall survival rate has still an extremely poor [1]. Although cigarette smoking is the major cause of lung cancer, only a small fraction of smokers develop this disease, suggesting that other causes, including genetic susceptibility, might contribute to the variation in individual lung cancer risk [2], [3]. Genetic susceptibility to environmental or occupational diseases is believed to play an important role in determining individual differences in the development of cancer. Research activities have focused on polymorphisms in DNA-repair genes as an important component of susceptibility because DNA-repair activities are critical for the protection of the genome and the prevention of cancer [4]. At the cellular level, checkpoints can be activated to arrest the cell cycle and transcription can be unregulated to compensate for the damage or the cell can apoptosis [5]. DNA repair is essential in protecting the cellular genome from environmental hazards, such as tobacco smoke [6]. Several studies have shown that a reduced DNA repair capacity is associated with increased lung cancer risk [7]–[9]. Many DNA repair genes carry genetic polymorphisms, with the potential to modulate gene function and alter DNA repair capacity [10].

The DNA repair pathways, including nucleotide excision repair (NER), base excision repair (BER) and double-strand break repair (DSBR) play an important role in repairing the DNA damage resulting from chemical alterations of a single base, such as methylated, oxidized, or reduced bases [11], [12]. The DNA repair enzymes *XRCC1* play a central role in the BER pathway [13], [14]. *XRCC1* is located on chromosome no. 19q13.2–13.3, and its gene product is implicated in single-strand break repair and base excision repair mechanisms [15]. *XRCC1* encodes a protein that function in the repair of single-strand breaks. Shen et al [16] identified three coding polymorphisms in the *XRCC*1 gene at codons 194 (Arg to Trp), 280 (Arg to His), and 399 (Arg to Gln). 5′UTR–77T>C is a novel polymorphism identified in the *XRCC1* gene located in the 5′untranslated region. Hao et al. [50] have reported that functional SNP −77T>C decreased transcriptional activity of C-allelecontaining promoter with higher affinity to Sp1 binding.

In the DSBR pathway, *XRCC*3 participate in DNA double-strand break/recombination repair and likely participates [17]–[19]. DSBs are the most common form of radiation-induced DNA damage [20] and are repaired by two pathways-homologous recombination repair (HRR) and no homologous end-joining [21]–[23]. The HRR pathway consists of at least 16 protein components, including XRCC3. A common polymorphism in exon 7 of the *XRCC3* gene results in an amino acid substitution at codon 241 (Thr241Met) that may affect the enzyme function and/or its interaction with other proteins involved in DNA damage and repair [24].

Molecular epidemiological studies have reported the association of *XRCC1* Arg399Gln, Arg194Trp, Arg280His, −77T>C, and *XRCC3* T241M with lung cancer risk [25]–[73], but the results remain conflicting rather than conclusive. Although several studies [81]–[86] previously performed pooling analyses regarding the association of XRCC1 Arg399Gln, Arg194Trp, Arg280His, −77T>C, and XRCC3 T241M with lung cancer risk. However, several published studies were not included in these meta-analyses and additional original studies with larger sample sizes have been published since then. Importantly, the previous meta-analyses on *XRCC1* Arg194Trp, Arg280His, and Arg399Gln with lung cancer risk have shown conflicting conclusions. Hence, the association of these polymorphic genes remains unknown. In order to explore the association between *XRCC1* Arg399Gln, Arg194Trp, Arg280His, −77T>C, and *XRCC3* T241M polymorphisms with lung cancer risk, a meta-analysis was conducted to summarize the data. Meta-analysis is a powerful tool for summarizing the different studies. It can not only overcome the problem of small size and inadequate statistical power of genetic studies of complex traits, but also provide more reliable results than a single case–control study.

## Materials and Methods

### Identification and eligibility of relevant studies

A comprehensive literature search was performed using the PubMed, ISI, and Embase databases for relevant articles published (last search was updated on Jan 12, 2013) with the following key words “*XRCC1*” or “*XRCC3*”, “polymorphism”, and “cancer” or “carcinoma” combined with “lung”. All eligible studies were retrieved, and their bibliographies were checked for other relevant publications. We excluded data that were unpublished or published in abstract only. We also reviewed the Cochrane Library for relevant articles. Additional articles were identified by hand searching references in the eligible articles and review articles that possibly have been missed in the initial search. Authors were contacted directly regarding crucial data not reported in original articles. When the same sample was used in several publications, only the study with the largest sample size was included following careful examination.

### Inclusion criteria

The included studies needed to have met the following criteria: (1) only the case–control studies were considered, (2) evaluated the *XRCC1* Arg399Gln, Arg194Trp, Arg280His, −77T>C, and *XRCC3* T241M polymorphisms and lung cancer risk, and (3) sufficient published data for estimating an odds ratio (OR) with 95% confidence interval (CI). Major reasons for exclusion of studies were as follows: (1) not cancer research, (2) only case population, (3) duplicate of previous publication, and (4) the distribution of genotypes among controls are not in Hardy–Weinberg equilibrium (*P*<0.01).

### Data extraction

Information was carefully extracted from all eligible studies independently by two investigators according to the inclusion criteria listed above. The following data were collected from each study: first author's name, year of publication, country of origin, ethnicity, source of controls, genotyping method, match, sample size, and numbers of cases and controls in the *XRCC1* Arg399Gln, Arg194Trp, Arg280His, −77T>C, and *XRCC3* T241M genotypes whenever possible. Ethnicity was categorized as “Caucasian”, “African”, and “Asian”. When a study did not state which ethnic groups were included or if it was impossible to separate participants according to phenotype, the sample was termed as “mixed population”. We did not define any minimum number of patients to include in this meta-analysis. Articles that reported different ethnic groups and different countries or locations, we considered them different study samples for each category cited above.

### Statistical analysis

Crude odds ratios (ORs) together with their corresponding 95% confidence intervals (95% CIs) were used to assess the strength of association between the *XRCC1* Arg399Gln, Arg194Trp, Arg280His, −77T>C, and *XRCC3* T241M polymorphisms and lung cancer risk. The pooled ORs were performed for dominant model (Arg399Gln: Arg/Gln+Gln/Gln vs. Arg/Arg, Arg194Trp: Arg/Trp+Trp/Trp vs. Arg/Arg, Arg280His: Arg/His+His/His vs. Arg/Arg, −77T>C: TC+CC vs. TT, and T241M: TM+MM vs. TT); recessive model (Arg399Gln: Arg/Gln+Arg/Arg vs. Gln/Gln, Arg194Trp: Arg/Trp+Arg/Arg vs. Trp/Trp, Arg280His: Arg/His+Arg/Arg vs. His/His, −77T>C: TC+TT vs. CC, and T241M: TM+TT vs. MM); additive model (Arg399Gln: Arg/Arg vs. Gln/Gln, Arg194Trp: Arg/Arg vs. Trp/Trp, Arg280His: Arg/Arg vs. His/His, −77T>C: TT vs. CC, and T241M: TT vs. MM), respectively. Between-study heterogeneity was assessed by calculating *Q*-statistic (Heterogeneity was considered statistically significant if *P*<0.10) [74] and quantified using the *I^2^* value, Venice criteria [75] for the *I^2^* test included: “*I^2^*<25% represents no heterogeneity, *I^2^* = 25–50% represents moderate heterogeneity, *I^2^* = 50–75% represents large heterogeneity, and *I^2^*>75% represents extreme heterogeneity”. If results were not heterogeneous, the pooled ORs were calculated by the fixed-effect model (we used the *Q*-statistic, which represents the magnitude of heterogeneity between-studies) [76]. Otherwise, a random-effect model was used (when the heterogeneity between-studies were significant) [77]. We also performed subgroup analyses by ethnicity (Caucasian and Asian), source of controls, histological type, gender, and smoking habits. Moreover, the extent to which the combined risk estimate might be affected by individual studies was assessed by consecutively omitting every study from the meta-analysis (leave-one-out sensitivity analysis). This approach would also capture the effect of the oldest or first positive study (first study effect). Secondly, we also ranked studies according to sample size, and then repeated this meta-analysis. Sample size was classified according to a minimum of 200 participants and those with fewer than 200 participants. The cite criteria were previously described [78]. We assessed Hardy–Weinberg equilibrium (HWE) for each study using the goodness-of-fit test (*χ* ^2^ or Fisher exact test) only in control groups, and deviation was considered when *P*<0.01. Begg's funnel plots [79] and Egger's linear regression test [80] were used to assess publication bias. If publication bias existed, the Duval and Tweedie non-parametric “trim and fill” method was used to adjust for it. A meta-regression analysis was carried out to identify the major sources of between-studies variation in the results, using the log of the ORs from each study as dependent variables, and ethnicity, source of controls, and sample size as the possible sources of heterogeneity. All of the calculations were performed using STATA version 10.0 (STATA Corporation, College Station, TX).

## Results

### Literature Search and Meta-analysis Databases

**Figure 1:**
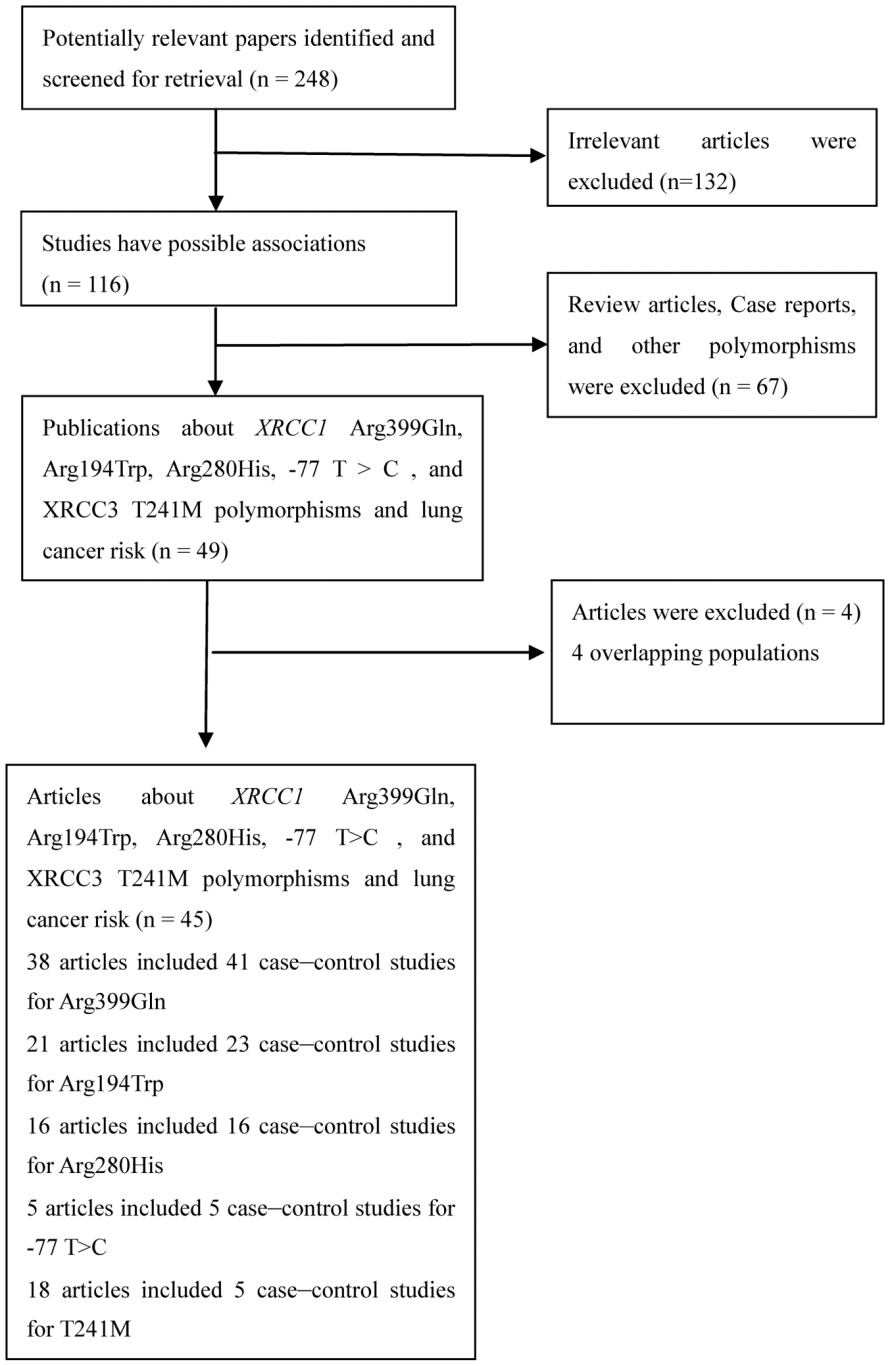
Study flow chart explaining the selection of the 45 eligible articles included in the meta-analysis.

Relevant publications were retrieved and preliminarily screened. As shown in Fig. 1, 248 publications were identified, among which 132 irrelevant papers were excluded. Thus, 116 publications were eligible. Among these publications, 67 articles were excluded because they were review articles, case reports, and other polymorphisms of *XRCC1* and *XRCC3*. In addition, Genotype distributions in the controls of all the eligible studies were in agreement with HWE. 4 articles [32], [37], [46], [60] were excluded because of their populations overlapped with another 2 included study [25], [33], [55], [59]. As summarized in Table 1, 45 articles with 104 case–control studies publications were selected in the final meta-analysis, including 14156 cases and 16,667 controls for *XRCC1* Arg399Gln (from 41 studies), 7,426 cases and 9,603 controls for Arg194Trp (from 23 studies), 6,211 cases and 6,763 controls for Arg280His (from 16 studies), 2,487 cases and 2,576 controls for −77T>C (from 5 studies), and 8,560 cases and 11,557 controls for *XRCC3* T241M (from 19 studies). Among these studies, five studies were included in the dominant model only because they provided the genotypes of TM+MM *versus* TT or Arg/Gln+Gln/Gln *versus* Arg/Arg as a whole and one study was included in the recessive model only because it provided the genotypes of TM+TT *versus* MM. 45 were population-based studies and 59 were hospital-based studies. 51 were conducted in Caucasians, 46 were conducted in Asians, and 6 studies were conducted in Africans. The remained were conducted in mixed ethnicity. Tables S1–S5 in File S1 listed ethnicity, HWE, and the numbers of cases and controls for *XRCC*1 Arg399Gln, Arg194Trp, Arg280His, −77T>C, and *XRCC3* T241M. All of the cases were pathologically confirmed.

**Table 1:** Main characteristics of all studies included in the meta-analysis.

First author/year	Country	Ethnicity	SNPS	GM	SC	Matching
Ratnasinghe [25] 2001	China	Asian	Arg399Gln	TaqMan	PB	Age/sex
Ratnasinghe [25] 2001	China	Asian	Arg194Trp	TaqMan	PB	Age/sex
Ratnasinghe [25] 2001	China	Asian	Arg280His	TaqMan	PB	Age/sex
David-Beabes [26] 2001	USA	Caucasian	Arg399Gln	PCR-RFLP	PB	Age/sex/ethnicity
David-Beabes [26] 2001	USA	Caucasian	Arg194Trp	PCR-RFLP	PB	Age/sex/ethnicity
David-Beabes [26] 2001	USA	African	Arg399Gln	PCR-RFLP	PB	Age/sex/ethnicity
David-Beabes [26] 2001	USA	African	Arg194Trp	PCR-RFLP	PB	Age/sex/ethnicity
David-Beabes [27] 2001	USA	Caucasian	T241M	PCR-RFLP	PB	Age/sex/ethnicity
David-Beabes [27] 2001	USA	African	T241M	PCR-RFLP	PB	Age/sex/ethnicity
Divine [28] 2001	USA	Caucasian	Arg399Gln	PCR-RFLP	HB	No clear
Chen [29] 2002	China	Asian	Arg399Gln	PCR	PB	Age/sex
Chen [29] 2002	China	Asian	Arg194Trp	PCR	PB	Age/sex
Park [30] 2002	Korea	Asian	Arg399Gln	PCR-RFLP	HB	Age
Misra [31] 2003	USA	Caucasian	Arg399Gln	PCR	PB	Age
Misra [31] 2003	USA	Caucasian	Arg280His	PCR	PB	Age
Misra [31] 2003	USA	Caucasian	T241M	PCR	PB	Age
Zhou [33] 2003	USA	Caucasian	Arg399Gln	PCR-RFLP	PB	No clear
Wang [34] 2003	USA	Mixed	T241M	PCR-RFLP	PB	Age/ethnicity/gender
Ito [35] 2004	Japan	Asian	Arg399Gln	PCR-CTTP	HB	Age/sex
Popanda [36] 2004	German	Caucasian	Arg399Gln	PCR	HB	No clear
Popanda [36] 2004	German	Caucasian	T241M	PCR	HB	No clear
Harms [38] 2004	German	Caucasian	Arg399Gln	PCR	HB	Age/ethnicity/gender
Harms [38] 2004	German	Caucasian	T241M	PCR	HB	Age/ethnicity/gender
Jacobsen [39] 2004	Danish	Caucasian	T241M	PCR	PB	No clear
Zhang [40] 2005	China	Asian	Arg399Gln	PCR-RFLP	PB	Age/sex
Hung [41] 2005	Europe	Caucasian	Arg399Gln	PCR	HB	Age/sex/area
Hung [41] 2005	Europe	Caucasian	Arg194Trp	PCR	HB	Age/sex/area
Hung [41] 2005	Europe	Caucasian	Arg280His	PCR	HB	No clear
Hung [41] 2005	Europe	Caucasian	T241M	PCR	HB	No clear
Vogel [42] 2004	Danish	Caucasian	Arg399Gln	PCR	PB	No clear
Vogel [42] 2004	Danish	Caucasian	Arg280His	PCR	PB	No clear
Schneider [43] 2005	German	Caucasian	Arg399Gln	PCR	HB	No clear
Schneider [43] 2005	German	Caucasian	Arg194Trp	PCR	HB	No clear
Schneider [43] 2005	German	Caucasian	Arg280His	PCR	HB	No clear
Shen [44] 2005	China	Asian	Arg399Gln	PCR	PB	Age/sex/village
Shen [44] 2005	China	Asian	Arg194Trp	PCR	PB	Age/sex/village
Shen [44] 2005	China	Asian	Arg280His	PCR	PB	Age/sex/village
Chan [45] 2005	China	Asian	Arg399Gln	PCR-RFLP	HB	Age/sex
Chan [45] 2005	China	Asian	Arg194Trp	PCR-RFLP	HB	Age/sex
Hu [47] 2005	China	Asian	Arg399Gln	PCR	HB	Age/sex/area
Hu [47] 2005	China	Asian	Arg194Trp	PCR	HB	Age/sex/area
Hu [47] 2005	China	Asian	−77T>C	PCR-RFLP	HB	Age/sex/area
Zienolddiny [48] 2006	Norway	Caucasian	Arg399Gln	TaqMan	PB	Age/sex/smoking
Zienolddiny [48] 2006	Norway	Caucasian	Arg194Trp	TaqMan	PB	Age/sex/smoking
Zienolddiny [48] 2006	Norway	Caucasian	Arg280His	TaqMan	PB	Age/sex/smoking
Zienolddiny [48] 2006	Norway	Caucasian	T241M	TaqMan	PB	Age/sex/smoking
Matullo [49]2006	Europe	Caucasian	Arg399Gln	TaqMan	PB	Age/sex/smoking
Matullo [49]2006	Europe	Caucasian	T241M	TaqMan	PB	Age/sex/smoking
Hao [50] 2006	China	Asian	Arg399Gln	PCR-RFLP	PB	Age/sex/ethnicity
Hao [50] 2006	China	Asian	Arg194Trp	PCR-RFLP	PB	Age/sex/ethnicity
Hao [50] 2006	China	Asian	Arg280His	PCR-RFLP	PB	Age/sex/ethnicity
Hao [50] 2006	China	Asian	−77T>C	PCR-RFLP	PB	Age/sex/ethnicity
Landi [51] 2006	Europe	Caucasian	Arg399Gln	PCR	HB	Age/sex/center/area
Landi [51] 2006	Europe	Caucasian	Arg194Trp	PCR	HB	Age/sex/center/area
Landi [51] 2006	Europe	Caucasian	Arg280His	PCR	HB	Age/sex/center/area
Landi [51] 2006	Europe	Caucasian	T241M	PCR	HB	Age/sex/center/area
Ryk [52] 2006	Swede	Caucasian	Arg399Gln	PCR	PB	Age/sex/smoking
Ryk [52] 2006	Swede	Caucasian	T241M	TaqMan	PB	Age/sex/smoking
De Ruyck [53] 2007	Belgium	Caucasian	Arg399Gln	PCR-RFLP	HB	Age/sex
De Ruyck [53] 2007	Belgium	Caucasian	Arg194Trp	PCR-RFLP	HB	Age/sex
De Ruyck [53] 2007	Belgium	Caucasian	Arg280His	PCR-RFLP	HB	Age/sex
De Ruyck [53] 2007	Belgium	Caucasian	−77T>C	PCR-RFLP	HB	Age/sex
Pachouri [54] 2007	India	Asian	Arg399Gln	PCR-RFLP	PB	No clear
Pachouri [54] 2007	India	Asian	Arg194Trp	PCR-RFLP	PB	No clear
Yin [55] 2007	China	Asian	Arg399Gln	PCR-RFLP	HB	Age/gender/ethnicity
Yin [55] 2007	China	Asian	Arg194Trp	PCR-RFLP	HB	Age/gender/ethnicity
Yin [55] 2007	China	Asian	Arg280His	PCR-RFLP	HB	Age/gender/ethnicity
Lopez-Cima [56] 2007	Spain	Caucasian	Arg399Gln	PCR-RFLP	HB	Age/gender/ethnicity
Lopez-Cima [56] 2007	Spain	Caucasian	T241M	PCR-RFLP	HB	Age/gender/ethnicity
Zhang [57] 2007	China	Asian	T241M	PCR	HB	No clear
Improta [58] 2008	Italy	Caucasian	Arg399Gln	PCR-RFLP	HB	Age/sex
Improta [58] 2008	Italy	Caucasian	Arg194Trp	PCR-RFLP	HB	Age/sex
Improta [58] 2008	Italy	Caucasian	T241M	PCR-RFLP	HB	Age/sex
Li [59] 2008	China	Asian	Arg399Gln	PCR-RFLP	HB	Age
Li [59] 2008	China	Asian	Arg194Trp	PCR-RFLP	HB	Age
Li [59] 2008	China	Asian	Arg280His	PCR-RFLP	HB	Age
Li [59] 2008	China	Asian	−77T>C	PCR-RFLP	HB	Age
Hsieh [61] 2009	China	Asian	−77T>C	PCR	HB	No clear
Cote [62] 2009	USA	Caucasian	Arg399Gln	PCR	PB	Age/race
Cote [62] 2009	USA	African	Arg399Gln	PCR	PB	Age/race
Qian [63] 2010	China	Asian	T241M	PCR	HB	Age/gender
Qian [63] 2010	China	Asian	Arg399Gln	PCR	HB	Age/gender
Tanaka [64] 2010	Japan	Asian	Arg194Trp	PCR	HB	Age/sex/ethnicity
Kim [65] 2010	Korea	Asian	Arg399Gln	PCR	HB	No clear
Kim [65] 2010	Korea	Asian	Arg280His	PCR	HB	No clear
Osawa [66] 2010	Japan	Asian	T241M	PCR-RFLP	HB	No clear
Osawa [66] 2010	Japan	Asian	Arg399Gln	PCR-RFLP	HB	No clear
Huang [67] 2011	China	Asian	T241M	PCR-RFLP	HB	Age/sex
Janik [68] 2011	Poland	Caucasian	Arg399Gln	PCR-SSCP	HB	Age/sex/smoking/diet
Janik [68] 2011	Poland	Caucasian	Arg194Trp	PCR-SSCP	HB	Age/sex/smoking/diet
Janik [68]2011	Poland	Caucasian	Arg280His	PCR-SSCP	HB	Age/sex/smoking/diet
Li [69] 2011	China	Asian	Arg399Gln	PCR-CTTP	HB	Age/sex/area
Wang [70]2012	China	Asian	Arg194Trp	PCR-RFLP	HB	Age/sex/area
Wang [70]2012	China	Asian	Arg280His	PCR-RFLP	HB	Age/sex/area
Wang [70]2012	China	Asian	Arg399Gln	PCR-RFLP	HB	Age/sex/area
Chikako [71] 2012	Japan	Asian	Arg399Gln	PCR-RFLP	HB	Age/sex
Chikako [71] 2012	Japan	Asian	T241M	PCR-RFLP	HB	Age/sex
Sreeja [72] 2008	India	Asian	Arg399Gln	PCR-RFLP	HB	Age/sex
Chang [73] 2009	USA	Caucasian	Arg399Gln	PCR-RFLP	PB	Age/sex/area
Chang [73] 2009	USA	Caucasian	Arg194Trp	PCR-RFLP	PB	Age/sex/area
Chang [73] 2009	USA	Caucasian	Arg280His	PCR-RFLP	PB	Age/sex/area
Chang [73] 2009	USA	African	Arg399Gln	PCR-RFLP	PB	Age/sex/area
Chang [73] 2009	USA	African	Arg194Trp	PCR-RFLP	PB	Age/sex/area

SNPS Single-nucleotide polymorphism studied, GM Genotyping method, SC Source of controls, SSCP Single-strand conformation polymorphism, CTPP Contronting two-pair primers, SNPS Single-nucleotide polymorphism studied, PB Population-based study, HB Hospital-based study.

### Quantitative synthesis

Table 2 listed the main results of the meta-analysis of *XRCC1* Arg399Gln polymorphism and lung cancer risk. When all the eligible studies were pooled into the meta-analysis of *XRCC1* Arg399Gln polymorphism, no significant association was found in any genetic model. However, significant between-study heterogeneity was detected in any genetic model. Hence, we performed subgroup analysis by ethnicity, histological type, smoker habits, gender, and source of controls. Among the stratified analysis, significantly increased lung cancer risk was observed in non-smokers (recessive model: OR = 1.57, 95% CI = 1.02–2.42, *P* value of heterogeneity test [*P*_h_] = 0.026, *I* ^2^ = 49.4%).

**Table 2:** Results of meta-analysis for XRCC1 and XRCC3 polymorphisms and lung cancer risk.

Variables	No. comparisons (SZ case/control)	Dominant model		Recessive model		Additive model	
		OR (95% CI)	*P*_h_/*I* ^2^	OR (95% CI)	*P*_h_/*I* ^2^	OR (95% CI)	*P*_h_/*I* ^2^
XRCC1 Arg399Gln							
Overall	41 (14,156/16,667)	1.00 (0.94–1.07)*	0.009/37.9%	1.05 (0.94–1.18)*	0.017/36.4%	1.05 (0.93–1.19)*	0.003/43.3%
Caucasian	19 (7,308/9,140)	0.98 (0.92–1.04)	0.560/0.0%	1.00 (0.87–1.16)*	0.054/38.4%	0.99 (0.89–1.10)	0.120/29.7%
Asian	19 (6,324/6,883)	1.06 (0.94–1.20)*	<0.001/60.5%	1.17 (0.96–1.41)*	0.038/40.7%	1.16 (0.93–1.46)*	0.002/56.3%
African	3 (524/644)	1.04 (0.81–1.35)	0.682/0.0%	0.79 (0.39–1.62)	0.603/0.0%	0.80 (0.39–1.64)	0.645/0.0%
PB	18 (5,943/7,925)	0.94 (0.87–1.01)	0.116/29.5%	1.01 (0.90–1.14)	0.145/27.1%	0.97 (0.86–1.10)	0.119/29.8%
HB	23 (8,213/8,742)	1.07 (0.98–1.16)*	0.062/33.4%	1.15 (0.98–1.34)*	0.015/44.7%	1.17 (0.98–1.40)*	0.004/50.9%
AC	11 (1,821/5,536)	1.13 (0.92–1.39)*	0.002/64.4%	1.31 (0.92–1.87)*	0.001/66.7%	1.34 (0.89–2.03)*	<0.001/73.3%
SC	6 (1,688/4,014)	0.97 (0.75–1.26)*	0.006/69.4%	1.06 (0.89–1.27)	0.225/29.5%	1.10 (0.77–1.57)*	0.058/56.1%
SCLC	3 (112/879)	0.75 (0.37–1.55)*	0.088/58.8%	0.67 (0.32–1.43)	0.642/0.0%	0.62 (0.28–1.37)	0.997/0.0%
Non-smokers	15 (1,300/2,874)	1.09 (0.83–1.43)*	0.001/62.4%	**1.57 (1.02**–**2.42)** *	0.026/49.4%	1.63 (0.99–2.68)*	0.007/57.6%
Smokers	16 (5,081/4,525)	1.02 (0.94–1.11)	0.536/0.0%	1.02 (0.89–1.18)	0.886/0.0%	1.04 (0.89–1.21)	0.743/0.0%
Male	3 (441/414)	1.08 (0.83–1.42)	0.559/0.0%	1.02 (0.39–4.31)*	0.087/65.8%	1.02 (0.60–1.72)	0.159/49.7%
Female	5 (773/826)	0.96 (0.58–1.60)*	0.001/78.1%	1.94 (0.73–5.14)*	0.014/71.8%	1.86 (0.55–6.27)*	0.001/80.6%
XRCC1 Arg194Trp							
Overall	23 (7,426/9,603)	0.96 (0.86–1.07)*	0.042/36.5%	**1.23 (1.05**–**1.44)**	0.216/18.8%	**1.22 (1.04**–**1.44)**	0.107/28.9%
Caucasian	10 (3,926/5,639)	0.88 (0.78–1.01)	0.472/0.0%	1.32 (0.78–2.23)	0.104/41.2%	1.29 (0.76–2.18)	0.102/41.5%
Asian	11 (3,091/3,441)	1.02 (0.88–1.19)*	0.065/42.6%	**1.22 (1.03**–**1.45)**	0.277/17.5%	**1.22 (1.02**–**1.45)**	0.111/36.0%
PB	11 (2,610/4,446)	0.98 (0.87–1.11)	0.356/9.2%	1.17 (0.93–1.49)	0.359/9.1%	1.16 (0.91–1.48)	0.264/19.5%
HB	12 (4,816/5,157)	0.94 (0.80–1.11)*	0.015/53.1%	**1.28 (1.03**–**1.59)**	0.141/32.2%	1.29 (0.90–1.86)*	0.077/40.8%
AC	5 (880/3,276)	0.97 (0.80–1.18)	0.634/0.0%	1.43 (0.86–2.40)	0.587/0.0%	1.41 (0.82–2.41)	0.682/0.0%
SC	3 (1,147/2,876)	0.86 (0.70–1.05)	0.850/0.0%	1.38 (0.68–2.80)	0.558/0.0%	1.35 (0.63–2.88)	0.554/0.0%
Non-smokers	7 (618/1,666)	1.07 (0.86–1.34)	0.242/24.6%	1.40 (0.38–5.17)*	0.030/62.8%	1.12 (0.26–4.79)*	0.031/66.1%
Smokers	6 (2,886/2,476)	**0.83 (0.71**–**0.98)**	0.141/39.7%	0.93 (0.44–1.99)	0.804/0.0%	0.74 (0.30–1.85)	0.681/0.0%
XRCC1 Arg280His							
Overall	16 (6,211/6,763)	1.04 (0.83–1.29)*	<0.001/74.7%	1.30 (0.71–2.37)*	0.065/39.3%	1.46 (0.99–2.15)	0.146/29.0%
Caucasian	9 (4,030/4,464)	1.06 (0.92–1.22)	0.133/35.6%	0.96 (0.50–1.87)	0.161/34.9%	1.37 (0.68–2.78)	0.721/0.0%
Asian	7 (2,181/2,299)	0.97 (0.64–1.48)*	<0.001/86.3%	1.48 (0.68–3.21)*	0.072/48.2%	1.45 (0.60–3.48)*	0.023/58.9%
PB	7 (2,247/2,683)	1.06 (0.90–1.23)	0.334/12.5%	1.32 (0.41–4.20)*	0.048/52.9%	1.43 (0.77–2.63)	0.274/20.4%
HB	9 (3,964/4,080)	0.93 (0.64–1.35)*	<0.001/84.1%	1.54 (0.94–2.51)	0.244/24.2%	1.32 (0.60–2.93)*	0.097/44.1%
AC	3 (795/2,864)	0.70 (0.34–1.42)*	0.001/85.5%	0.36 (0.07–2.01)	–	0.25 (0.04–1.39)	–
Non-smokers	6 (715/1,340)	0.63 (0.35–1.13)*	0.001/74.7%	0.78 (0.29–2.12)	0.606/0.0%	0.64 (0.23–1.75)	0.380/0.0%
Smokers	6 (2,977/2,457)	1.04 (0.78–1.38)	0.942/0.0%	3.93 (0.44–35.3)	0.820/0.0%	4.16 (0.46–37.6)	0.851/0.0%
XRCC1 −77T>C (rs3213245)							
Overall	5 (2,487/2,576)	**1.45 (1.27**–**1.66)**	0.638/0.0%	**1.73 (1.14**–**2.62)**	0.469/0.0%	**1.91 (1.24**–**2.94)**	0.494/0.0%
XRCC3 T241M (rs861539)							
Overall	19 (8,560/11,557)	0.93 (0.83–1.04)*	0.011/48.8%	1.09 (0.88–1.35)*	0.003/56.8%	1.06 (0.83–1.37)*	<0.001//65.5%
Caucasian	12 (6,089/8,992)	0.92 (0.80–1.06)*	0.003/60.6%	1.07 (0.84–1.36)*	0.001/67.6%	1.04 (0.79–1.37)*	<0.001/72.5%
Asian	5 (2,201/2,141)	0.96 (0.78–1.18)	0.447/0.0%	1.20 (0.60–2.39)	0.504/0.0%	1.18 (0.54–2.55)	0.302/16.5%
PB	8 (1,528/2,950)	0.91 (0.79–1.04)	0.623/0.0%	0.95 (0.75–1.20)	0.820/0.0%	0.93 (0.73–1.19)	0.775/0.0%
HB	11 (7,032/8,607)	0.96 (0.80–1.14)*	0.002/66.1%	1.25 (0.88–1.77)*	<0.001/72.3%	1.22 (0.81–1.85)*	<0.001/78.6%
Smokers	5 (698/756)	0.83 (0.67–1.03)	0.137/42.8%	1.32 (0.81–2.14)	0.749/0.0%	1.22 (0.74–2.01)	0.822/0.0%

AC Adenocarcinoma, SC Squamous cell carcinoma, SCLC Small cell lung cancer,

* Random-effect model was used when *P* value of heterogeneity test (*P*_h_)<0.10; otherwise, fixed-effect model was used.

Table 2 also listed the main results of the meta-analysis of *XRCC1* Arg194Trp polymorphism and lung cancer risk. When all the eligible studies were pooled into the meta-analysis of *XRCC1* Arg194Trp polymorphism, significantly increased risk of lung cancer was observed in the recessive model (OR = 1.23, 95% CI = 1.05–1.44, *P*_h_ = 0.216, *I^2^* = 18.8%) and additive model (OR = 1.22, 95% CI = 1.04–1.44, *P*_h_ = 0.107, *I^2^* = 28.9%). Among the stratified analyses, significantly increased lung cancer risk was observed in Asians (recessive model: OR = 1.22, 95% CI = 1.03–1.45, *P*_h_ = 0.277, *I^2^* = 17.5%; additive model: OR = 1.22, 95% CI = 1.02–1.45, *P*_h_ = 0.111, *I^2^* = 36.0%) and the hospital-based controls (recessive model: OR = 1.28, 95% CI = 1.03–1.59, *P*_h_ = 0.141, *I^2^* = 32.2%).

Table 2 also listed the main results of the meta-analysis of *XRCC1* Arg280His polymorphism and lung cancer risk. When all the eligible studies were pooled into the meta-analysis of *XRCC1* Arg280His polymorphism, no significant association was observed in any genetic model. In the stratified analyses, there was not still significant association between *XRCC1* Arg280His polymorphism and lung cancer risk.

**Figure 2:**
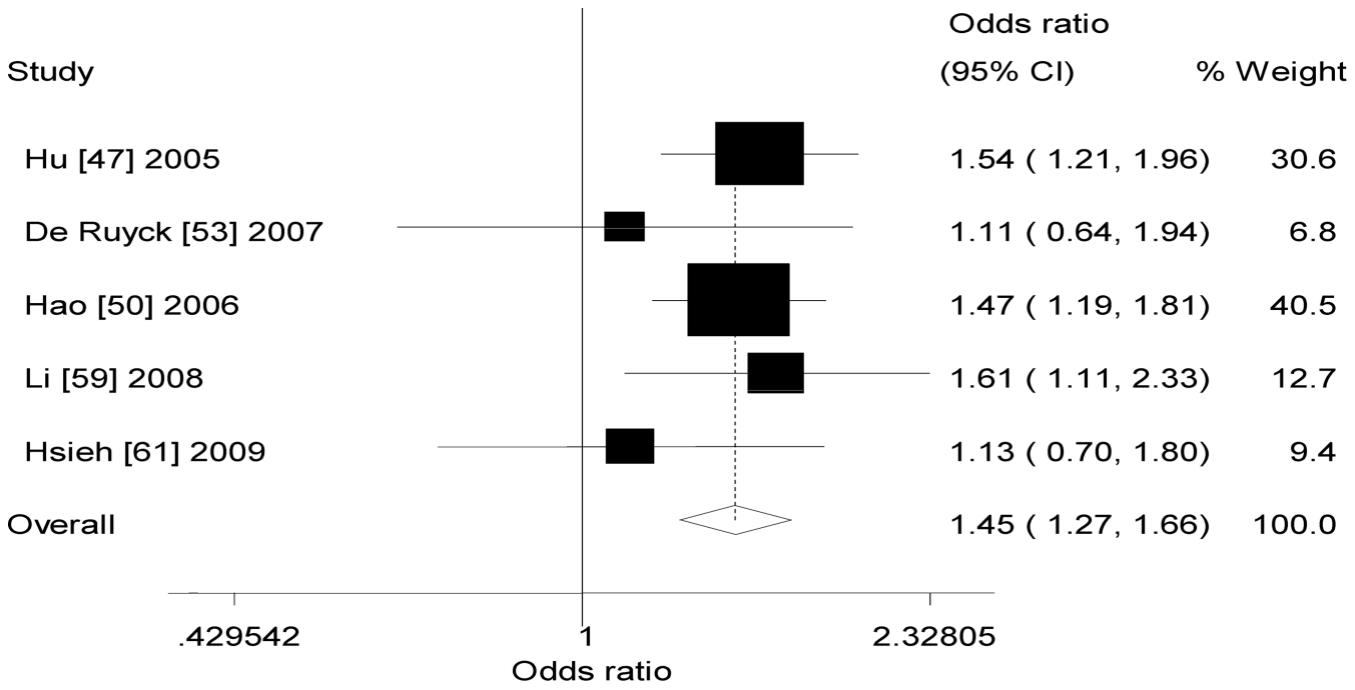
Forest plot of XRCC1 −77T>C polymorphism and lung cancer risk (dominant model).

**Figure 3:**
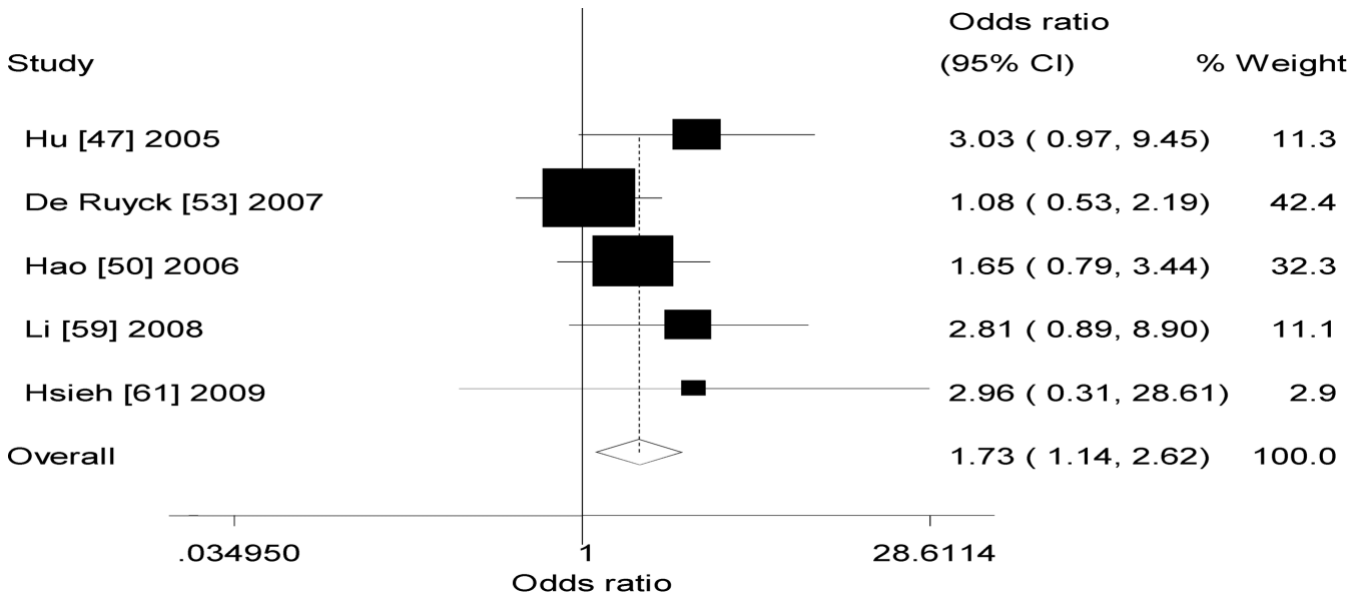
Forest plot of XRCC1 −77T>C polymorphism and lung cancer risk (recessive model).

**Figure 4:**
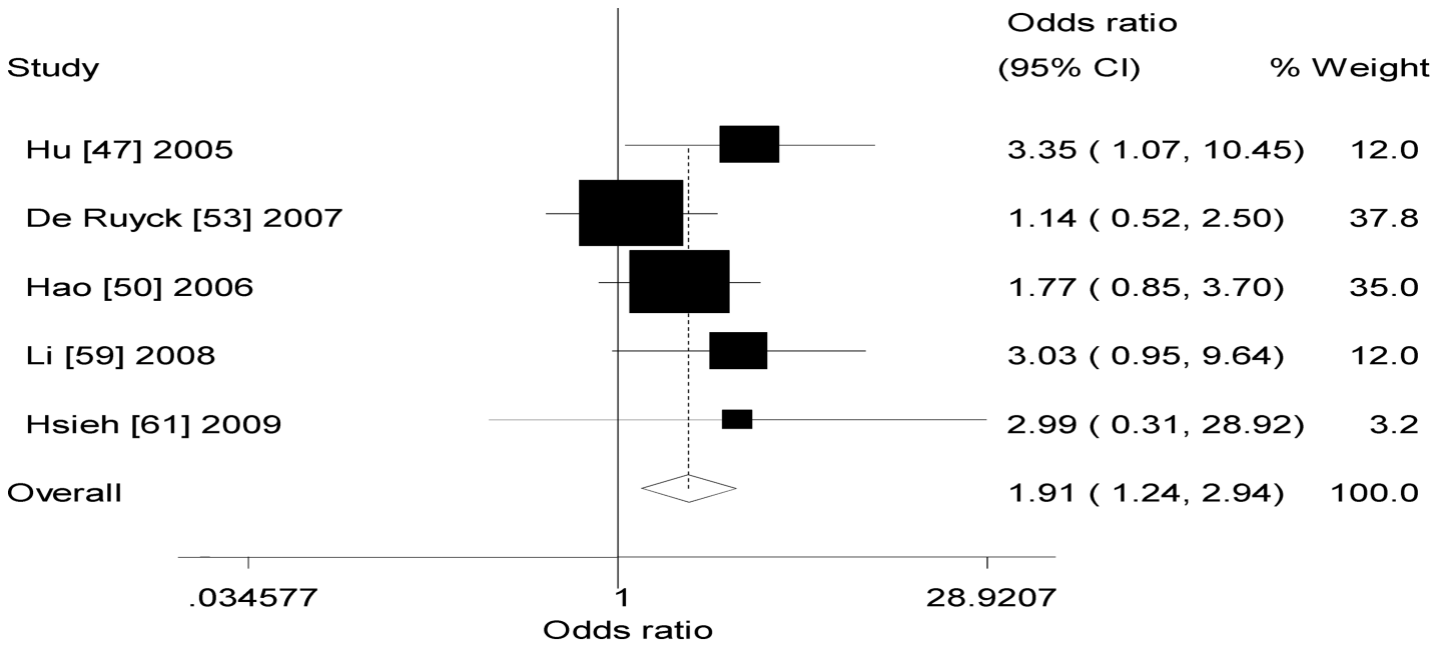
Forest plot of XRCC1 −77T>C polymorphism and lung cancer risk (additive model).

Table 2 also listed the main results of the meta-analysis of *XRCC1* −77T>C polymorphism and lung cancer risk. When all the eligible studies were pooled into the meta-analysis of *XRCC1* −77 T>C polymorphism, significant increased risk of lung cancer was observed in any genetic model (dominant model: OR = 1.45, 95% CI = 1.27–1.66, *P*_h_ = 0.638, *I^2^* = 0.0%, Fig. 2; recessive model: OR = 1.73, 95% CI = 1.14–2.62, *P*_h_ = 0.469, *I^2^* = 0.0%, Fig. 3; additive model: OR = 1.91, 95% CI = 1.24–2.94, *P*_h_ = 0.494, *I^2^* = 0.0%, Fig. 4).

Table 2 also listed the main results of the meta-analysis of XRCC3 T241M polymorphism and lung cancer risk. When all the eligible studies were pooled into the meta-analysis of XRCC3 T241M polymorphism, there was no evidence of significant association between lung cancer risk and XRCC3 T241M polymorphism in any genetic model. In the stratified analysis, there was not still significant association.

### Heterogeneity and sensitive analysis

**Figure 5:**
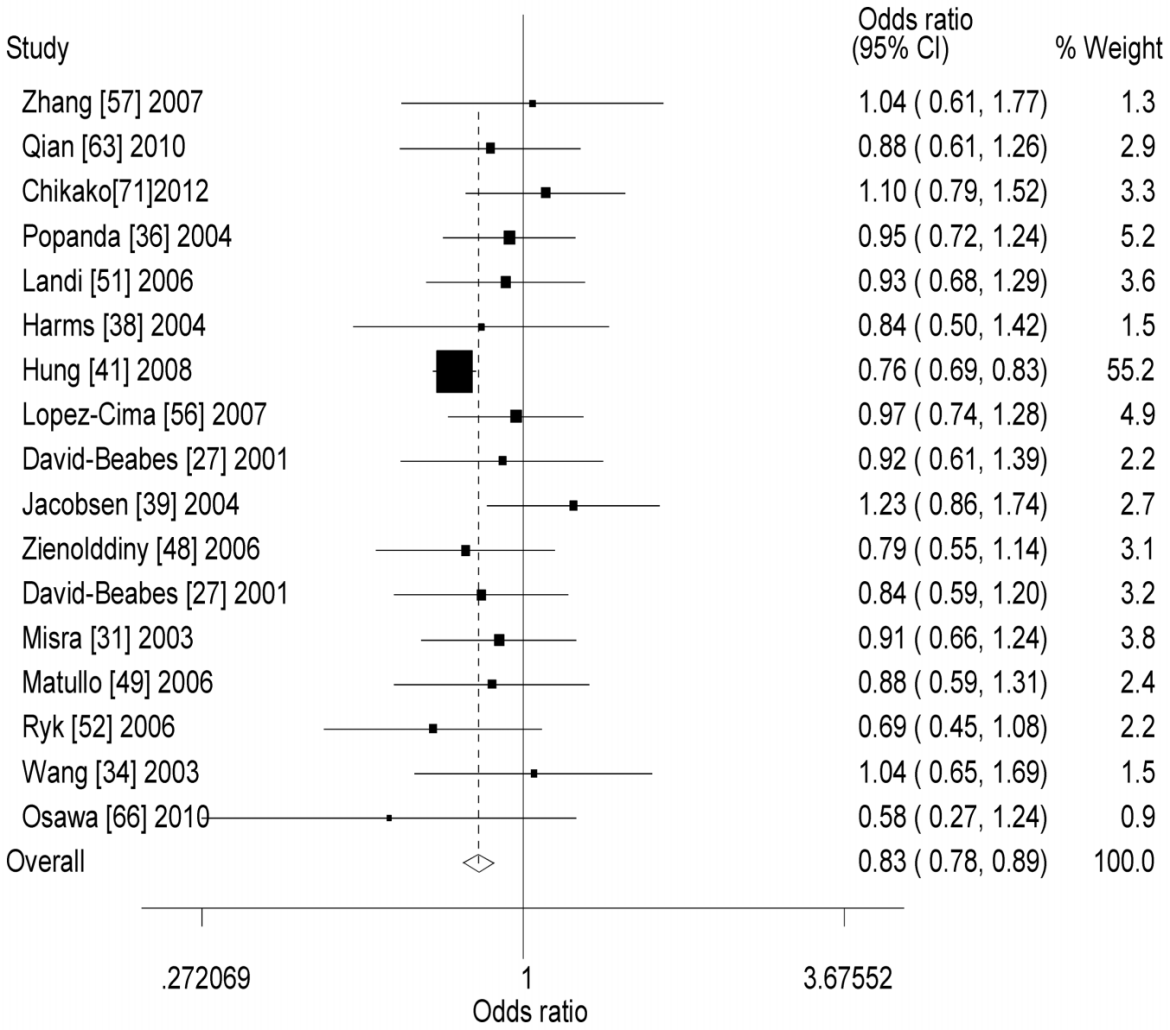
Forest plot of XRCC3 T241M polymorphism and lung cancer risk when one study was excluded (dominant model).

There was significant heterogeneity among these studies for dominant model comparison (*XRCC1* Arg399Gln: *P*_h_ = 0.009, *XRCC1* Arg194Trp: *P*_h_ = 0.042, *XRCC1* Arg280His: *P*_h_<0.001, and *XRCC3* T241M: *P*_h_ = 0.011); recessive model comparison (*XRCC1* Arg399Gln: *P*_h_ = 0.017 and *XRCC3* T241M = 0.003); additive model comparison (*XRCC1* Arg399Gln: *P*_h_ = 0.003 and *XRCC*3 T241M<0.001). Then, we assessed the source of heterogeneity by meta-regression analysis. We found that source of controls, ethnicity, and sample size did not contribute to substantial heterogeneity among the meta-analysis (data not shown). Sensitivity analyses were conducted to determine whether modification of the inclusion criteria of this meta-analysis affected the results. Although the sample size for cases and controls in all eligible studies ranged from 100 to 8,488, the corresponding pooled ORs were not qualitatively altered with or without the study of small sample. However, for *XRCC1* Arg399Gln polymorphism, when one study was excluded, the results were changed in non-smokers (recessive model: OR = 1.12, 95% CI = 0.96–1.21, *P*_h_ = 0.114, *I^2^* = 32.6%). For *XRCC1* Arg194Trp polymorphism, when one study was excluded, the results were also changed in overall analysis (recessive model: OR = 1.17, 95% CI = 0.99–1.39, *P*_h_ = 0.313, *I^2^* = 11.4%; additive model: OR = 1.15, 95% CI = 0.97–1.37, *P*_h_ = 0.227, *I^2^* = 18.3%), Asians (recessive model: OR = 1.16, 95% CI = 0.97–1.38, *P*_h_ = 0.447, *I^2^* = 0.0%; additive model: OR = 1.14, 95% CI = 0.95–1.37, *P*_h_ = 0.295, *I^2^* = 16.1%), hospital-based studies (recessive model: OR = 1.17, 95% CI = 0.92–1.49, *P*_h_ = 0.241, *I^2^* = 21.9%), and smokers (dominant model: OR = 0.87, 95% CI = 0.74–1.03, *P*h = 0.409, *I*2 = 0.0%). For *XRCC3* T241M polymorphism, when one study was excluded, significantly decreased lung cancer risk was observed in overall analysis (dominant model: OR = 0.83, 95% CI = 0.78–0.89, *P*_h_ = 0.302, *I* ^2^ = 13.0%, Fig. 5; recessive model: OR = 0.90, 95% CI = 0.81–1.00, *P*_h_ = 0.507, *I* ^2^ = 0.0%; additive model: OR = 0.82, 95% CI = 0.74–0.92, *P*_h_ = 0.278, *I* ^2^ = 16.1%), Caucasians (dominant model: OR = 0.82, 95% CI = 0.76–0.87, *P*_h_ = 0.248, *I* ^2^ = 20.5%; recessive model: OR = 0.89, 95% CI = 0.80–0.99, *P*_h_ = 0.427, *I* ^2^ = 6.3%; additive model: OR = 0.81, 95% CI = 0.73–0.91, *P*_h_ = 0.277, *I* ^2^ = 18.1%), and hospital-based controls (dominant model: OR = 0.81, 95% CI = 0.76–0.88, *P*_h_ = 0.193, *I* ^2^ = 28.2%; recessive model: OR = 0.89, 95% CI = 0.79–1.00, *P*_h_ = 0.213, *I* ^2^ = 25.9%; additive model: OR = 0.80, 95% CI = 0.71–0.90, *P*_h_ = 0.108, *I* ^2^ = 40.6%).

### Publication bias

**Figure 6:**
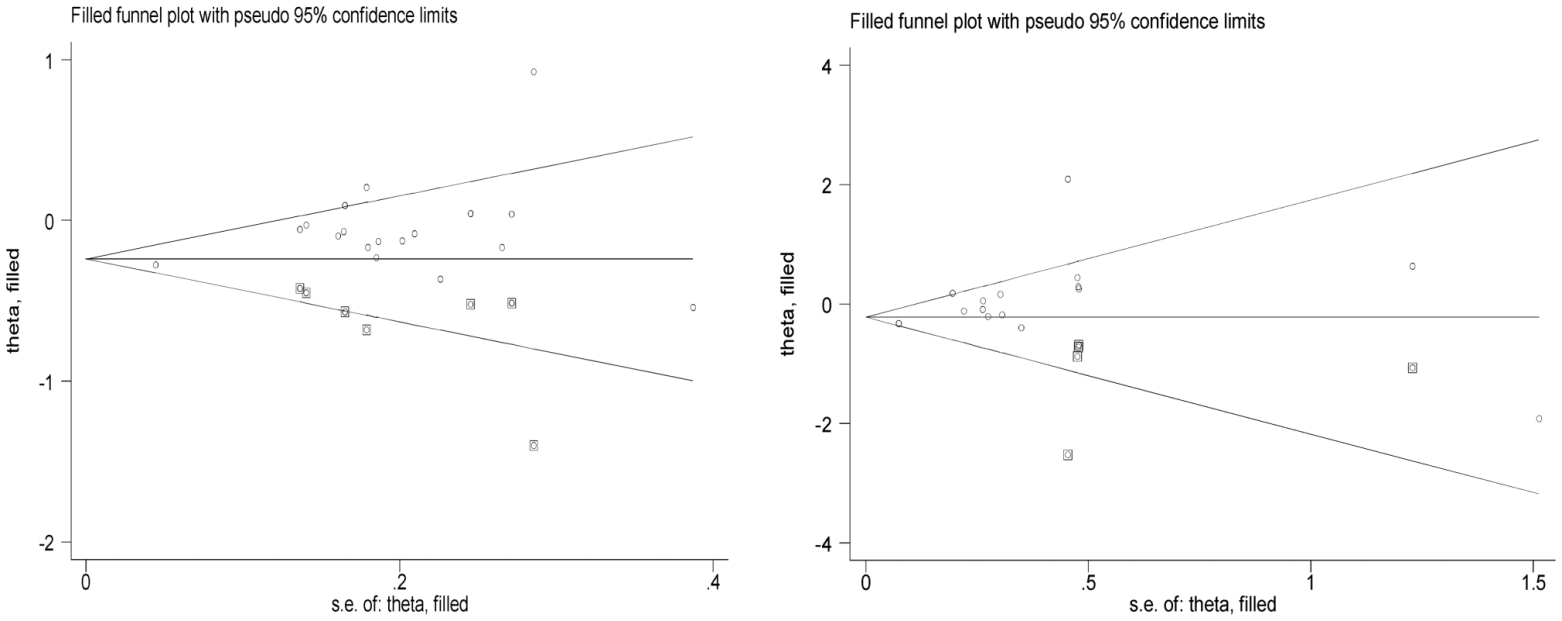
The Duval and Tweedie nonparametric “trim and fill” method's funnel plot funnel plot of the meta-analysis of lung cancer risk and XRCC3 T241M polymorphism (dominant model and additive model).

Begg's funnel plot and Egger's test did not reveal any publication bias for *XRCC1* Arg399Gln (*P* = 0.546 for dominant model, *P* = 0.767 for recessive model, and *P* = 0.984 for additive model), Arg194Trp (*P* = 0.588 for dominant model, *P* = 0.416 for recessive model, *P* = 0.555 for additive model), Arg280His (*P* = 0.439 for dominant model, *P* = 0.520 for recessive model, *P* = 0.292 for additive mode), −77T>C (P = 0.186 for dominant model, P = 0.162 for recessive model, P = 0.246 for additive mode), although possible publication bias was suggested between *XRCC3* T241M polymorphism and lung cancer risk in dominant model (*P* = 0.012) and additive model (*P* = 0.041). This might be a limitation for this meta-analysis because studies with null findings, especially those with small sample size, are less likely to be published. The Duval and Tweedie non-parametric “trim and fill” method was used to adjust for publication bias. Meta-analysis with and without “trim and fill” did not draw different conclusion (Fig. 6), indicating that our results were statistically robust.

## Discussion

BER and DSBR play an important role in repairing the DNA damage resulting from chemical alterations of a single base, such as methylated, oxidized, or reduced bases. BER includes two major processes (excision of damaged base residues and core BER reaction, including strand incision at the abasic site, one nucleotide gap-filling reaction, and sealing of the remaining nick). It is well known that a number of proteins are involved in these steps, of which XRCC1 play key roles. XRCC1 acts as a facilitator or coordinator in BER, through its interaction with poly (ADP-ribose) polymerase, DNA polymerase b, and DNA ligase III [15], [95]. Four coding polymorphisms were identified in the XRCC1 gene at the codons 194 (Arg to Trp), 280 (Arg to His), 399 (Arg to Gln), and −77 T>C. Whereas the functional effects of these polymorphisms in XRCC1 have not been well known, amino acid changes at evolutionary conserved regions may alter its function. In particular, the 399Gln polymorphism resulting from a guanine to adenine nucleotide occurs in the poly (ADP-ribose) polymerase binding domain and may affect complex assembly or repair efficiency. The XRCC3 gene codes for a protein involved in homologous recombinational repair (HRR) for double strand breaks of DNA (DBSs) and cross-link repair in mammalian cells [20]. During HRR, the XRCC3 protein interacts with Rad51 protein and likely contributes to maintain chromosome stability. A common polymorphism in exon 7 of the XRCC3 gene results in an amino acid substitution at codon 241 (Thr241Met) that may affect the enzyme function and/or its interaction with other proteins involved in DNA damage and repair [20]. Many molecular epidemiological studies have reported the role of *XRCC1* Arg399Gln, Arg194Trp, Arg280His, −77T>C, and *XRCC3* T241M in lung cancer risk [25]–[73], but the results remain conflicting rather than conclusive. In order to resolve this conflict, meta-analysis was performed to examine the association between *XRCC1* and *XRCC3* polymorphisms and lung cancer risk, by critically reviewing 41 studies on *XRCC1* Arg399Gln, 23 studies on Arg194Trp, 16 studies on Arg280His, 5 studies on −77T>C, and 19 studies on *XRCC3* T241M.

Overall, our meta-analysis indicates that *XRCC1* −77T>C polymorphism is associated with increased lung cancer risk when all eligible studies were pooled into the meta-analysis. In further stratified and sensitivity analyses, significantly decreased lung cancer risk was observed in Caucasians for XRCC3 T241M, but not in Asians. It should be considered that the apparent inconsistency of these results may underlie differences in ethnicity, lifestyle and disease prevalence as well as possible limitations due to the relatively small sample size. The current knowledge of carcinogenesis indicates a multi-factorial and multistep process that involves various genetic alterations and several biological pathways. Thus, it is unlikely that risk factors of cancer work in isolation from each other. And the same polymorphisms may play different roles in cancer susceptibility, because cancer is a complicated multi-genetic disease, and different genetic backgrounds may contribute to the discrepancy. And even more importantly, the low penetrance genetic effects of single polymorphism may largely depend on interaction with other polymorphisms and/or a particular environmental exposure.

Present meta-analysis results were not consistent with a previous meta-analysis [81]–[86] on *XRCC1* and *XRCC3* polymorphisms with lung cancer risk. Kiyohara et al. [81] included 18 case–control studies on XRCC1 Arg399Gln, 9 studies on Arg194Trp, and 7 studies on Arg280His. Their results suggested that *XRCC1* Arg399Gln polymorphism was associated with increased lung cancer risk among Asians (OR = 1.34, 95% CI = 1.16–1.54) and Arg194Trp and Arg280His polymorphisms were not associated with lung cancer risk. However, at any case, their results about Arg399Gln and lung cancer risk essentially remains an open field in Asians, as the number of studies (n = 6) is considerably smaller than that needed for the achievement of robust conclusions [96]. Wang et al. [82] included 30 case–control studies on XRCC1 Arg399Gln and 16 studies on Arg194Trp. Their results indicated that certain XRCC1 codon 399 and 194 variant may affect the susceptibility of lung cancer. Dai et al. [83] included 39 studies on XRCC1 Arg399Gln, 22 studies on Arg194Trp, and 12 studies on Arg280His. Their meta-analysis had demonstrated that codon 194, codon 399 and −77 T>C polymorphisms of XRCC1 gene might have contributed to individual susceptibility to lung cancer. However, in further subgroup and sensitivity analyses, we found XRCC1 Arg399Gln and Arg194Trp polymorphisms were not associated with lung cancer risk when one study was excluded, hence, we thought XRCC1 Arg399Gln and Arg194Trp polymorphisms may be not associated with lung cancer risk. Sun et al. [84] in 2010 included 14 case–control studies on XRCC3 T241M, their meta-analysis found that there was no evidence showing a significant association between *XRCC3* Thr241Met polymorphism and lung cancer risk. Zhan et al. [85] in 2013 included 17 case–control studies on XRCC3 T241M, their meta-analysis indicated that there was no evidence showing a significant correlation between XRCC3 Thr241Met polymorphism and lung cancer risk stratified analysis by ethnicity, histology and smoking status. Xu et al. [86] in 2013 included 17 case–control studies on XRCC3 T241M, their meta-analysis all available data did not support any appreciable association between the XRCC3 Thr241Met polymorphism and lung cancer risk in any populations. However, in further subgroup and sensitivity analyses, we found XRCC3 T241M polymorphism was associated with lung cancer risk in Caucasians. Vineis et al. [97] in 2009 only included 3 case–control studies on XRCC1 polymorphism, their found XRCC1 −77T>C polymorphism was associated with lung cancer risk. Having analyzed an almost twofold larger number of studies than the previous meta-analysis [81]–[86], our results seem to confirm and establish the trend in the meta-analysis of *XRCC1* Arg399Gln, Arg194Trp, Arg280His, −77T>C, and T241M polymorphisms that the data by the previous meta-analysis [81]–[86] had indicated. Importantly, we carefully performed sensitivity analysis according to sample size and leave-one-out analysis, conducted different conclusions with the previous meta-analysis. For *XRCC1* −77 T>C polymorphism, the T to C mutation greatly enhances the affinity of nuclear protein Sp1 to the *XRCC1* promoter region, which may inhibit its transcription [50]. Up to now, only five case–control studies were conducted the association between −77 T>C polymorphism and lung cancer risk [47], [50], [53], [59], [61]. The pooled OR of these five studies, comparing the combined variant genotype CT+CC to wild genotype TT, was 1.45 (95% CI 1.27–1.66). Among these five studies, four studies carried out in Asians with large sample size all showed that −77 T>C polymorphism was significantly associated with increased risk of developing lung cancer and the summary OR was 1.48 (95% CI 1.28–1.70), which suggested that the −77 T>C polymorphism may be contributed to the developing of lung cancer in Asians. Due to the relative small sample size from the selected studies, a case–control study with larger sample size or multiple center study will be needed to get conclusive results. In addition, the sample size was also too small for the *XRCC3* Thr241Met polymorphism and lung cancer risk in Asians and Africans. Hence, a case–control study with larger sample size or multiple center study will be needed to get conclusive results in Asians and Africans.

DNA repair is well known as a “double-edged sword” in cancer studies. Epidemiological evidence supports that DNA repair capacity is one of the determinants of genetic susceptibility to cancer [87]–[89]. Liu et al. [98] found that XRCC1 −77T>C polymorphism may be a genetic determinant for developing breast cancer. However, other cancer such as gastric cancer, colorectal cancer and so on with XRCC1 −77T>C polymorphism remained unclear. Hence, some new studies are needed to get conclusive results among other cancer. On the other hand, tumors with enhanced DNA repair capacity would exhibit an intrinsic resistance to the anti-tumour activity during chemotherapy and radiotherapy [90]. Fluorouracil (5-FU)/oxaliplatin-based chemotherapy induced DNA damages and causes cell death [91]. These damages are mainly repaired by the BER pathway. A 5-fold greater incidence of failure by 5-FU/oxaliplatin therapy had been reported for metastatic colorectal cancer patients with *XRCC1* R399Q (QQ or QR) substitution compared with that of the RR genotype, suggesting that the polymorphism was associated with resistance to oxaliplatin/5-FU therapy [92]. In addition, it had been found that *XRCC1* codon 194 variant was having a significant protective effect on development of late radiotherapy reactions in normal tissue [93]. It had also found that interactions among XRCC1 codon 194 variant was associated with sensitivity to platinum-based chemotherapy [100]. Furthermore, Sak et al. [94] indicated that high levels of *XRCC1* protein expression were associated with improved cancer-specific survival in patients following radical radiotherapy. However, Liu et al. [99] indicated that XRCC1 T-77C could not be genetic determinant for prognosis of advanced non-small-cell lung cancer (NSCLC) patients treated with platinum-based chemotherapy. Therefore, *XRCC1* Arg399Gln and Arg194Trp might play important roles in the drug sensitivity during chemotherapy and radio sensitivity during radiotherapy and XRCC1 −77 T>C may be not play important roles in the drug sensitivity during chemotherapy.

Heterogeneity is a potential problem when interpreting the results of all meta-analyses. As looked through our study carefully, we found that the study of Improta et al. [58] was noted to be a major source of heterogeneity for *XRCC3* T241M and Li et al. [59] was also noted to be a major source of heterogeneity for *XRCC1* Arg280His. The reason may be that the study of Improta et al. [58] and Li et al. [59] were hospital-based studies. Importantly, when Improta et al. [58] was excluded, significantly decreased lung cancer risk was observed in overall analysis, Caucasians, and hospital-based controls.

Some limitations of this meta-analysis should be addressed. First, misclassifications on disease status and genotypes may influence the results, because cases in some studies were not confirmed by pathology or other gold standard method, and the quality control of genotyping was also not well-documented in some studies. Second, in the subgroup analysis may have had insufficient statistical power to check an association, Third, our results were based on unadjusted estimates, while a more precise analysis should be conducted if individual data were available, which would allow for the adjustment by other co-variants, including environmental factors and other lifestyle. In spite of these, our meta-analysis also had some advantages. First, it provides pooled data on a substantial number of cases and controls and increased statistical power of the analysis. Second, although possible publication bias was suggested between *XRCC3* T241M polymorphism and lung cancer risk, adjusting for possible publication bias using the Duval and Tweedie nonparametric “trim and fill” method showed that the results did not change, indicating that the whole pooled results should be unbiased. Third, our meta-analysis explores and analyzes the sources of heterogeneity between studies about XRCC3 T241M and Arg280His in lung cancer risk.

In conclusion, this meta-analysis indicates that *XRCC1* −77T>C shows an increased lung cancer risk and *XRCC3* T241M polymorphism is associated with decreased lung cancer risk in Caucasians. However, a study with a larger sample size is needed to further evaluate gene-environment interaction on *XRCC1* Arg399Gln, Arg194Trp, Arg280His, −77T>C, and *XRCC3* T241M polymorphisms and lung cancer risk.

## Supporting Information

Checklist S1 **PRISMA 2009 checklist.**
(DOC)

File S1 **Table S1, Genotypes, p values and subset of cases of XRCC1 Arg399Gln polymorphism included in the meta-analysis. Table S2, Genotypes, p values and subset of cases of XRCC1 Arg194Trp (rs1799782) polymorphism included in the meta-analysis. Table S3, Genotypes, P values and subset of cases of XRCC1 Arg280His (rs25489) polymorphism included in the meta-analysis. Table S4, Genotypes, P values and subset of cases of XRCC1 −77T>C (rs3213245) polymorphism included in the meta-analysis. Table S5, Genotypes, P values and subset of cases of XRCC3 T241M (rs861539) polymorphism included in the meta-analysis.**
(DOC)
