# Supplementary material for: Association between XRCC1 and XRCC3 Polymorphisms with Lung Cancer Risk: A Meta-Analysis from Case-Control Studies
Source: PLoS One. 2013 Aug 26;8(8):e68457. doi: 10.1371/journal.pone.0068457 (PMC3753326; doi:10.1371/journal.pone.0068457)
Supplement: File S1 — Table S1, Genotypes, p values and subset of cases of XRCC1 Arg399Gln polymorphism included in the meta-analysis. Table S2, Genotypes, p values and subset of cases of XRCC1 Arg194Trp (rs1799782) polymorphism included in the meta-analysis. Table S3, Genotypes, P values and subset of cases of XRCC1 Arg280His (rs25489) polymorphism included in the meta-analysis. Table S4, Genotypes, P values and subset of cases of XRCC1 −77T>C (rs3213245) polymorphism included in the meta-analysis. Table S5, Genotypes, P values and subset of cases of XRCC3 T241M (rs861539) polymorphism included in the meta-analysis. (DOC) [file pone.0068457.s002.doc]

**Table S1** Genotypes, p values and subset of cases of XRCC1 Arg399Gln polymorphism included in the meta-analysis.

| First author/year | Ethnicity | Genotype distribution | | | | | | HWE |
| --- | --- | --- | --- | --- | --- | --- | --- | --- |
|  |  | Case | | | Control | | |  |
|  |  | Arg/Arg | Arg/Gln | Gln/Gln | Arg/Arg | Arg/Gln | Gln/Gln |  |
| Ratnasinghe ^[25]^ 2001 | Asian | 59 | 40 | 8 | 117 | 80 | 11 | 0.854 |
| David-Beabes ^[26]^ 2001 | Caucasian | 87 | 76 | 17 | 186 | 217 | 58 | 0.912 |
| David-Beabes ^[26]^ 2001 | African | 105 | 46 | 3 | 164 | 70 | 9 | 0.905 |
| Divine ^[28]^ 2001 | Caucasian | 82 | 61 | 29 | 65 | 64 | 14 | 0.954 |
| Chen ^[29]^ 2002 | Asian | 55 | 43 | 5 | 52 | 40 | 7 | 0.983 |
| Park ^[30]^ 2002 | Asian | 100 | 75 | 17 | 81 | 48 | 6 | 0.946 |
| Misra ^[31]^ 2003 | Caucasian | 151 | 140 | 24 | 154 | 130 | 29 | 0.978 |
| Zhou ^[33]^ 2003 | Caucasian | 467 | 468 | 156 | 551 | 546 | 143 | 0.918 |
| Ito ^[34]^ 2004 | Asian | 98 | 66 | 14 | 253 | 169 | 26 | 0.948 |
| Popanda ^[36]^ 2004 | Caucasian | 186 | 214 | 63 | 171 | 222 | 67 | 0.933 |
| Harms ^[38]^ 2004 | Caucasian | 59 | 42 | 9 | 56 | 55 | 8 | 0.524 |
| Vogel ^[42]^ 2004 | Caucasian | 117 | 104 | 35 | 108 | 121 | 40 | 0.812 |
| Zhang ^[40]^ 2005 | Asian | 535 | 363 | 102 | 531 | 380 | 89 | 0.213 |
| Hung ^[41]^ 2005 | Caucasian | 844 | 951 | 254 | 874 | 881 | 260 | 0.278 |
| Schneider ^[43]^ 2005 | Caucasian | 199 | 198 | 49 | 264 | 280 | 78 | 0.961 |
| Shen ^[44]^ 2005 | Asian | 72 | 40 | 4 | 54 | 51 | 4 | 0.155 |
| Chan ^[45]^ 2005 | Asian | 40 | 31 | 4 | 90 | 61 | 11 | 0.987 |
| Hu ^[47]^ 2005 | Asian | 378 | 284 | 48 | 370 | 282 | 58 | 0.92 |
| Zienolddiny ^[48]^ 2006 | Caucasian | 129 | 171 | 31 | 151 | 186 | 54 | 0.963 |
| Matullo ^[49]^ 2006 | Caucasian | 51 | 58 | 7 | 484 | 482 | 128 | 0.879 |
| Hao ^[50]^ 2006 | Asian | 566 | 376 | 82 | 585 | 432 | 101 | 0.263 |
| Landi ^[51]^ 2006 | Caucasian | 118 | 143 | 34 | 123 | 149 | 42 | 0.959 |
| Ryk ^[52]^ 2006 | Caucasian | 77 | 100^a^ |  | 59 | 94^a^ |  | NA |
| De Ruyck ^[53]^ 2007 | Caucasian | 38 | 53 | 18 | 46 | 50 | 13 | 0.995 |
| Pachouri ^[54]^ 2007 | Asian | 53 | 38 | 12 | 35 | 70 | 17 | 0.16 |
| Yin ^[55]^ 2007 | Asian | 138 | 65 | 2 | 132 | 52 | 9 | 0.437 |
| Lopez-Cima ^[56]^ 2007 | Caucasian | 222 | 219 | 75 | 217 | 234 | 82 | 0.362 |
| Improta ^[58]^ 2008 | Caucasian | 42 | 41 | 11 | 53 | 61 | 7 | 0.146 |
| Li ^[59]^ 2008 | Asian | 168 | 139 | 43 | 201 | 123 | 26 | 0.502 |
| Cote ^[62]^ 2009 | Caucasian | 172 | 159 | 56 | 160 | 200 | 46 | 0.375 |
| Cote ^[62]^ 2009 | African | 86 | 23 | 6 | 88 | 28 | 5 | 0.368 |
| Qian ^[63]^ 2010 | Asian | 321 | 221 | 39 | 332 | 236 | 35 | 0.716 |
| Kim ^[65]^ 2010 | Asian | 81 | 51 | 7 | 145 | 62 | 10 | 0.608 |
| Osawa ^[66]^ 2010 | Asian | 47 | 57^a^ |  | 61 | 59^a^ |  | NA |
| Janik ^[68]^ 2011 | Caucasian | 64 | 24 | 0 | 51 | 28 | 0 | 0.551 |
| Li ^[69]^ 2011 | Asian | 236 | 193 | 26 | 220 | 196 | 27 | 0.152 |
| Wang ^[70]^ 2012 | Asian | 105 | 83 | 21 | 137 | 96 | 23 | 0.587 |
| Chikako ^[71]^ 2012 | Asian | 243 | 171 | 48 | 242 | 121 | 16 | 0.984 |
| Sreeja ^[72]^ 2008 | Asian | 78 | 86 | 47 | 102 | 80 | 29 | 0.428 |
| Chang ^[73]^ 2009 | Caucasian | 54 | 47 | 12 | 155 | 127 | 16 | 0.206 |
| Chang ^[73]^ 2009 | African | 182 | 69 | 4 | 209 | 65 | 6 | 0.423 |

HWE Hardy–Weinberg equilibrium, Arg/Arg indicates wild-type, Arg/Gln indicates heterozygote, Gln/Gln indicates variant homozygote, NO nested. ^a^ For these just presenting the information for genotypes of Ser/Ser and Ser/Cys + Cys/Cys, dominant model is calculated only, NA Not available.

**Table S2** Genotypes, p values and subset of cases of XRCC1 Arg194Trp (rs1799782) polymorphism included in the meta-analysis.

| First author/year | Ethnicity | Genotype distribution | | | | | | HWE |
| --- | --- | --- | --- | --- | --- | --- | --- | --- |
|  |  | Case | | | Control | | |  |
|  |  | Arg/Arg | Arg/Trp | Trp/Trp | Arg/Arg | Arg/Trp | Trp/Trp |  |
| Ratnasinghe ^[25]^ 2001 | Asian | 52 | 47 | 9 | 85 | 104 | 21 | 0.410 |
| David-Beabes ^[26]^ 2001 | Caucasian | 158 | 22 | 0 | 407 | 54 | 0 | 0.814 |
| David-Beabes ^[26]^ 2001 | African | 142 | 10 | 2 | 205 | 36 | 2 | 0.981 |
| Chen ^[29]^ 2002 | Asian | 48 | 44 | 11 | 57 | 40 | 5 | 0.833 |
| Hung ^[41]^ 2005 | Caucasian | 1,878 | 259 | 10 | 1,828 | 292 | 12 | 0.996 |
| Schneider ^[43]^ 2005 | Caucasian | 389 | 53 | 4 | 544 | 75 | 3 | 0.989 |
| Shen ^[44]^ 2005 | Asian | 65 | 41 | 12 | 64 | 40 | 8 | 0.881 |
| Chan ^[45]^ 2005 | Asian | 50 | 22 | 3 | 79 | 67 | 16 | 0.947 |
| Hu ^[47]^ 2005 | Asian | 335 | 311 | 64 | 339 | 308 | 63 | 0.843 |
| Zienolddiny ^[48]^ 2006 | Caucasian | 309 | 26 | 1 | 368 | 35 | 2 | 0.506 |
| Matullo ^[49]^ 2006 | Caucasian | 98 | 16 | 2 | 951 | 141 | 2 | 0.393 |
| Hao ^[50]^ 2006 | Asian | 524 | 409 | 91 | 572 | 459 | 87 | 0.929 |
| Landi ^[51]^ 2006 | Caucasian | 263 | 32 | 1 | 262 | 53 | 1 | 0.639 |
| De Ruyck ^[53]^ 2007 | Caucasian | 101 | 8 | 1 | 93 | 17 | 0 | 0.861 |
| Pachouri ^[54]^ 2007 | Asian | 40 | 39 | 24 | 52 | 47 | 23 | 0.128 |
| Yin ^[55]^ 2007 | Asian | 120 | 98 | 23 | 119 | 109 | 21 | 0.850 |
| Improta ^[58]^ 2008 | Caucasian | 78 | 9 | 7 | 104 | 17 | 0 | 0.875 |
| Li ^[59]^ 2008 | Asian | 184 | 136 | 30 | 196 | 133 | 21 | 0.969 |
| Tanaka ^[64]^ 2010 | Asian | 28 | 15 | 7 | 25 | 23 | 2 | 0.513 |
| Janik ^[68]^ 2011 | Caucasian | 74 | 14 | 0 | 69 | 10 | 0 | 1.000 |
| Wang ^[70]^2012 | Asian | 89 | 80 | 40 | 138 | 90 | 28 | 0.088 |
| Chang ^[73]^ 2009 | Caucasian | 89 | 23 | 1 | 223 | 66 | 10 | 0.364 |
| Chang ^[73^] 2009 | African | 221 | 34 | 0 | 248 | 31 | 1 | 0.675 |

HWE Hardy–Weinberg equilibrium, Arg/Arg indicates wild-type, Arg/Trp indicates heterozygote, Trp/Trp indicates variant homozygote.

**Table S3** Genotypes, *P* values and subset of cases of XRCC1 Arg280His (rs25489) polymorphism included in the meta-analysis.

| First author/year | Ethnicity | Genotype distribution | | | | | | HWE |
| --- | --- | --- | --- | --- | --- | --- | --- | --- |
|  |  | Case | | | Control | | |  |
|  |  | Arg/Arg | Arg/His | His/His | Arg/Arg | Arg/His | His/His |  |
| Ratnasinghe ^[25]^ 2001 | Asian | 83 | 20 | 3 | 177 | 32 | 0 | 0.811 |
| Misra ^[31]^ 2003 | Caucasian | 260 | 47 | 2 | 260 | 42 | 0 | 0.805 |
| Hung ^[41]^ 2005 | Caucasian | 1,901 | 181 | 6 | 1,896 | 190 | 6 | 0.868 |
| Vogel ^[42]^ 2004 | Caucasian | 229 | 26 | 1 | 241 | 28 | 0 | 0.883 |
| Schneider ^[43]^ 2005 | Caucasian | 404 | 40 | 2 | 562 | 60 | 0 | 0.844 |
| Shen ^[44]^ 2005 | Asian | 76 | 30 | 5 | 81 | 28 | 1 | 0.698 |
| Zienolddiny ^[48]^ 2006 | Caucasian | 296 | 31 | 2 | 350 | 24 | 3 | 0.013 |
| Hao ^[50]^ 2006 | Asian | 848 | 169 | 7 | 904 | 204 | 10 | 0.598 |
| Landi ^[51]^ 2006 | Caucasian | 260 | 32 | 1 | 290 | 25 | 0 | 0.918 |
| De Ruyck ^[53]^ 2007 | Caucasian | 105 | 4 | 0 | 96 | 14 | 0 | 0.898 |
| Yin ^[55]^ 2007 | Asian | 190 | 46 | 2 | 179 | 59 | 4 | 0.936 |
| Li ^[59]^ 2008 | Asian | 266 | 79 | 5 | 74 | 72 | 4 | 0.02 |
| Kim ^[65]^ 2010 | Asian | 107 | 33 | 3 | 180 | 33 | 1 | 0.974 |
| Janik ^[68]^ 2011 | Caucasian | 66 | 22 | 0 | 67 | 12 | 0 | 0.886 |
| Wang ^[70]^2012 | Asian | 136 | 53 | 20 | 184 | 61 | 11 | 0.145 |
| Chang ^[73]^ 2009 | Caucasian | 242 | 51 | 5 | 86 | 25 | 1 | 0.122 |

HWE Hardy–Weinberg equilibrium, Arg/Arg indicates wild-type, Arg/His indicates heterozygote, His/His indicates variant homozygote.

**Table S4** Genotypes, *P* values and subset of cases of XRCC1 –77T>C (rs3213245) polymorphism included in the meta-analysis.

| First author | Ethnicity | Genotype distribution | | | | | | HWE |
| --- | --- | --- | --- | --- | --- | --- | --- | --- |
|  |  | Case | | | Control | | |  |
|  |  | TT | TC | CC | TT | TC | CC |  |
| Hu ^[47]^ 2005 | Asian | 500 | 198 | 12 | 558 | 148 | 4 | 0.216 |
| De Ruyck ^[53]^ 2007 | Caucasian | 37 | 53 | 19 | 40 | 52 | 18 | 0.987 |
| Hao ^[50]^ 2006 | Asian | 783 | 223 | 18 | 924 | 182 | 12 | 0.669 |
| Li ^[59]^ 2008 | Asian | 264 | 75 | 11 | 291 | 55 | 4 | 0.955 |
| Hsieh ^[61]^ 2009 | Asian | 251 | 40 | 3 | 250 | 37 | 1 | 0.975 |

HWE Hardy–Weinberg equilibrium, TT indicates wild-type, TC indicates heterozygote, CC indicates variant homozygote.

**Table S5** Genotypes, *P* values and subset of cases of XRCC3 T241M (rs861539) polymorphism included in the meta-analysis.

| First author/year | Ethnicity | Genotype distribution | | | | | | HWE |
| --- | --- | --- | --- | --- | --- | --- | --- | --- |
|  |  | Case | | | Control | | |  |
|  |  | TT | TM | MM | TT | TM | MM |  |
| David-Beabes ^[27]^ 2001 | African | 95 | 54 | 9 | 136 | 88 | 10 | 0.658 |
| David-Beabes ^[27]^ 2001 | Caucasian | 76 | 78 | 24 | 175 | 210 | 68 | 0.994 |
| Misra ^[31]^ 2003 | Caucasian | 160 | 124 | 29 | 149 | 134 | 23 | 0.632 |
| Wang ^[34]^ 2003 | Mixed | 69 | 43^a^ |  | 119 | 71^a^ |  | NA |
| Popanda ^[36]^ 2004 | Caucasian | 175 | 201 | 86 | 168 | 222 | 69 | 0.952 |
| Harms ^[38]^ 2004 | Caucasian | 61 | 37 | 12 | 61 | 49 | 9 | 0.981 |
| Jacobsen ^[39]^ 2004 | Caucasian | 95 | 123 | 38 | 113 | 113 | 43 | 0.268 |
| Zienolddiny ^[48]^ 2006 | Caucasian | 114 | 90 | 16 | 115 | 111 | 24 | 0.931 |
| Matullo ^[49]^ 2006 | Caucasian | 44 | 56 | 16 | 383 | 544 | 167 | 0.519 |
| Landi ^[51]^ 2006 | Caucasian | 127 | 132 | 36 | 129 | 143 | 40 | 0.999 |
| Improta ^[58]^ 2008 | Caucasian | 31 | 33 | 30 | 67 | 46 | 8 | 1.000 |
| Lopez-Cima ^[56]^ 2007 | Caucasian | 168 | 185 | 50 | 178 | 196 | 60 | 0.874 |
| Zhang ^[57]^ 2007 | Asian | 259 | 30 | 2 | 244 | 28 | 1 | 1.000 |
| Ryk ^[52]^ 2006 | Caucasian | 79 | 96^a^ |  | 56 | 98^a^ |  | NA |
| Osawa ^[66]^ 2010 | Asian | 92 | 12^a^ |  | 98 | 22^a^ |  | NA |
| Huang ^[67]^ 2011 | Asian | 759^b^ |  | 4 | 763^b^ |  | 3 | 0.540 |
| Qian ^[63]^ 2010 | Asian | 521 | 60 | 0 | 533 | 67 | 3 | 0.875 |
| Chikako^[71]^ 2012 | Asian | 352 | 97 | 13 | 295 | 77 | 7 | 0.756 |
| Hung ^[41]^ 2005 | Caucasian | 1,719 | 1,386 | 362 | 2,144 | 2,248 | 629 | 0.634 |

HWE Hardy–Weinberg equilibrium, TT indicates wild-type, TM indicates heterozygote, MM indicates variant homozygote, ^a^ For these just presenting the information for genotypes of TT and TC + CC, dominant model is calculated only, ^b^ For these just presenting the information for genotypes of CC and TT + TC, recessive model is only calculated.
